# Pathophysiology of respiratory failure in patients with osteogenesis imperfecta: a systematic review

**DOI:** 10.1080/07853890.2021.1980819

**Published:** 2021-09-27

**Authors:** S. Storoni, S. Treurniet, D. Micha, M. Celli, M. Bugiani, J. G. van den Aardweg, E. M. W. Eekhoff

**Affiliations:** a Section Endocrinology, Department of Internal Medicine, Amsterdam Bone Center, Amsterdam University Medical Center, Amsterdam, The Netherlands; b Department of Human Genetics, Amsterdam Movement Sciences, Amsterdam University Medical Center, Amsterdam, The Netherlands; c Department of Rare Bone Metabolism Center, Pediatric Department, Sapienza University of Rome, Rome, Italy; d Department of Pathology, Amsterdam University Medical Centre, Amsterdam, The Netherlands; e Department of Respiratory Medicine, Amsterdam University Medical Center, Amsterdam, The Netherlands

**Keywords:** Osteogenesis Imperfecta, respiratory mechanics, pulmonary function, lung pathophysiology, thoracic skeletal changes

## Abstract

**Introduction:**

Respiratory failure is a major cause of death in patients with Osteogenesis Imperfecta. Moreover, respiratory symptoms seem to have a dramatic impact on their quality of life. It has long been thought that lung function disorders in OI are mainly due to changes in the thoracic wall, caused by bone deformities. However, recent studies indicate that alterations in the lung itself can also undermine respiratory health.

**Objectives:**

Is there any intrapulmonary alteration in Osteogenesis Imperfecta that can explain decreased pulmonary function? The aim of this systematic literature review is to investigate to what extent intrapulmonary or extrapulmonary thoracic changes contribute to respiratory dysfunction in Osteogenesis Imperfecta.

**Methods:**

A literature search (in PubMed, Embase, Web of Science, and Cochrane), which included articles from inception to December 2020, was performed in accordance with the PRISMA guidelines.

**Results:**

Pulmonary function disorders have been described in many studies as secondary to scoliosis or to thoracic skeletal deformities. The findings of this systematic review suggest that reduced pulmonary function can also be caused by a primary pulmonary problem due to intrinsic collagen alterations.

**Conclusions:**

Based on the most recent studies, the review indicates that pulmonary defects may be a consequence of abnormal collagen type I distorting the intrapulmonary structure of the lung. Lung function deteriorates further when intrapulmonary defects are combined with severe thoracic abnormalities. This systematic review reveals novel findings of the underlying pathological mechanism which have clinical and diagnostic implications for the assessment and treatment of pulmonary function disorders in Osteogenesis Imperfecta.

**KEY MESSAGES**
- Decreased pulmonary function in Osteogenesis Imperfecta can be attributed to primary pulmonary defects due to intrapulmonary collagen alterations and not solely to secondary problems arising from thoracic skeletal dysplasia.
- Type I collagen defects play a crucial role in the development of the lung parenchyma and defects, therefore, affect pulmonary function. More awareness is needed among physicians about pulmonary complications in Osteogenesis Imperfecta to develop novel concepts on clinical and diagnostic assessment of pulmonary functional disorders.

## At a glance

### Scientific knowledge on the topic

Acute and chronic respiratory failure are major causes of death in patients with Osteogenesis Imperfecta. Aberrant type I collagen causes bone demineralization, deformities, and fractures, which in turn results in structural abnormalities of the chest wall and spine, limiting the pulmonary function. Treatment of Osteogenesis Imperfecta focuses on preventing fractures and correcting bone deformities, but the pulmonary disease is often ignored until breathing problems become severe. Type I collagen is found in many connective tissues, including the interstitial parenchyma of the mammalian lung. The question that arises is to what extent abnormal collagen contributes to the decreased pulmonary function of patients with OI. We performed a systematic review to examine all available knowledge on the pathogenesis of lung disease in OI.

### What this study adds to the field

Type I collagen defects play a crucial role in the development of the lung parenchyma and defects, therefore, affect pulmonary function. These findings will hopefully lead to more awareness among physicians about pulmonary complications in OI and novel concepts on clinical and diagnostic assessment of pulmonary disorders. We suggest pulmonary function and lung diffusion testing in OI patients even in the absence of thoracic malformations.

## Introduction

Osteogenesis Imperfecta (OI) is a rare inheritable condition commonly caused by mutations in genes (*COL1A1* and *COL1A2*) encoding collagen type I which is essential for healthy bone formation. Clinically, OI is primarily characterized by bone fragility, small stature, skeletal deformity, ligament laxity, blue sclerae, dentinogenesis imperfecta, hearing impairment, and cardiopulmonary disease. According to the Sillence classification OI type I is the least severe form and is mostly characterized by an insufficient level of synthesized collagen leading to a limited number of fractures. OI type II, III, and IV are mainly characterized by structural alterations in type I collagen; OI type II is perinatally lethal, OI type III is characterized by progressive deformations, multiple fractures, very short stature, and wheelchair dependency and type IV is relatively mild with a variable number of fractures [1–6].

Although bone fragility is the most common patient issue, extraskeletal manifestations also play a crucial role in the lives of patients with OI. Respiratory conditions are clinically often ignored until breathing problems become severe, even though the sparse literature on this topic supports that OI patients suffer from restrictive lung disease with dyspnoea and impaired pulmonary ventilation. Spirometry has been used as a primary measure to evaluate pulmonary function in OI, showing increasingly restrictive lung disease in correlation with the severity of OI. These patients are more vulnerable to viral and bacterial infections of upper and lower airways, chronic infection, and the formation of bronchiectasis [7–13].

The clinical urgency of lung disease in OI is also evident by the fact that respiratory failure appears to contribute significantly to morbidity and decreased quality of life in OI patients [14–17]. A cohort study in Denmark showed that OI patients have a subhazard ratio for death by the respiratory disease which is three times higher than in the reference population [15]. In OI type II many case reports describe perinatal death related to pulmonary hypoplasia and respiratory failure [18–22]. Moreover, the study of Yonko et al. on the quality of life in OI concluded that respiratory symptoms negatively impact psychosocial well-being and limit daily physical activity. These limitations occur regardless of their OI type, age, or scoliosis and reflect the dramatic impact of respiratory status on the quality of life for people with OI [16].

Type I collagen is a major constituent of the connective tissue of the lungs which is particularly found in bronchi, blood vessels, and the alveolar interstitium. Lung function greatly depends on collagen and elastin, which provide elastic strength to facilitate respiratory mechanics. Collagen type I defects in OI can therefore affect the lung structure and function [23–28].

All these data underline the necessity to examine collagen defects as an intrinsic factor in the aetiology of lung problems in OI patients, based on which clinical guidelines can be adjusted to improve critical points in the respiratory care of OI. This systematic literature review aims to investigate to what extent intrapulmonary or extrapulmonary thoracic wall changes contribute to respiratory dysfunction in OI patients in an effort to raise awareness about its pathophysiology and unmet clinical needs.

## Methods

The literature was systematically reviewed to identify publications on lung pathogenesis in Osteogenesis Imperfecta. This systematic review is reported in accordance with the Preferred Reporting Items for Systematic Reviews and Meta-Analyses (PRISMA)-statement. To identify all relevant publications, systematic searches were conducted in the bibliographic databases PubMed, Embase.com, Web of Science, and Cochrane from inception up to 7 December 2020, in collaboration with a medical information specialist.

Search terms included controlled terms from MeSH in PubMed and Emtree in Embase.com in addition to free-text terms. The following terms were used (including synonyms and closely related words): “Osteogenesis imperfecta”, “Brittle bone dis*”, “Respiratory insufficiency”, “Pulmonary function”, “Respiration/pathophysiology”. The search was performed without date or language restriction. Full search strategies for all databases can be found in the Supplementary Material.

### Study selection

**Figure 1.**
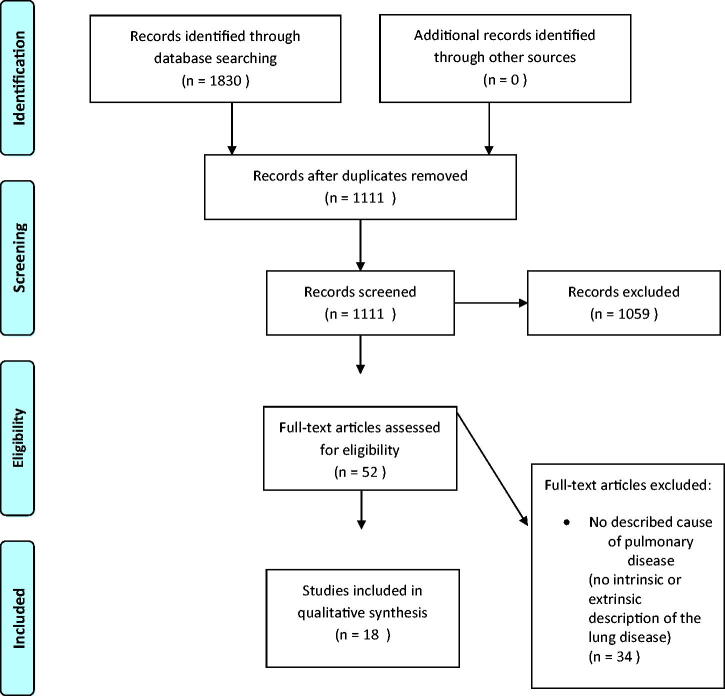
Flow diagram of the systematic review following the PRISMA guidelines.

After deduplication, a total of 1111 papers were identified. First, all titles were screened for eligibility by two researchers (SS and EE) after which all remaining abstracts were screened. In case of disagreement, the consensus was reached through dialogue. A total of 52 studies were included for full-text analysis. Thirty-four studies were excluded (Figure 1).

### Inclusion criteria

The following inclusion criteria were used: (1) studies published in English; (2) studies including patients with Osteogenesis Imperfecta; (3) studies adequately describing the cause of the lung problem; (4) studies describing OI in children, adults, or mice; (5) studies published as an original article; (6) full-text availability; and (7) all types of study design.

### Exclusion criteria

The following exclusion criteria were used: (1) studies published before 1970; (2) Systematic reviews; (3) Cole Carpenter syndrome; and (4) Bruck syndrome.

### Quality assessment

Quality assessment was performed on all included articles describing lung problems. The Quality assessment was independently performed by two researchers (SS and EE). In case of disagreement, the consensus was reached through dialogue. Most of the articles consisted of case-control, cohort, cross-sectional studies, and case series (82%). These articles were assessed using the Study Quality Assessment Tool created by NHLBI. The remaining 18% consisted of case reports. The quality of the reports was assessed according to the method described by Murad et al. [29].

## Results

### Intrapulmonary disease in OI mouse models

To date, four studies have evaluated lung parenchyma changes in OI mice [11,30–32]. These studies on animal models of OI, representing different OI types and genetic causes, all share post-natal primary defects in the lung parenchyma that are reminiscent of emphysematous changes (Table 1). In all studies, a diffuse increase in alveolar airway space and a loss of the alveolar walls was described. In three of these studies, the mean linear intercept length (Lm) was used to quantify the size of distal airspaces in OI and wild-type (WT) mice. There was a significant increase in the Lm value of the lungs of OI mice compared to the control mice. These alterations caused changes in the breathing pattern of the mice and affected their respiratory mechanics [11,32].

**Table 1.** Summary of mouse model characteristics.

Authors	Mouse Model	OI Phenotype	Analyzed tissue	Lung and other significant abnormalities	Authors supported pathophysiology
Baglole et al. (2018)	Col1a1Jrt/+ mice (number = 6) and wild-type littermates (number = 6). Col1a1Jrt/+: dominant mutation leading to collagen type I α1–chain alteration.	Model of severe dominant OI.	Lungs tissue and diaphragmatic histology.	Significant increase (+27%) in the mean linear intercept length (Lm) value in lungs of all OI mice compared to their Wild Type controls. Substantial decrease in the diaphragmatic thickness in the OI group (reduction of ∼28%). Reduced muscle mass and intrinsic contractile weakness of the diaphragm.	Intrapulmonary: emphysematous changes Extra-pulmonary: diaphragm weakness
Baldridge et al. (2010)	Crtap^−/−^ mice and wild type littermates. Lack of a functional prolyl 3-hydroxylation complex in the endoplasmic reticulum, which is essential for type I collagen post-translational modification and folding (number not reported).	Model of recessively inherited OI forms.	Lungs, kidneys, testes, and skin from the upper back histology.	Diffuse increase in alveolar airway space often accompanied by a thinning of the alveolar walls. This became more evident in the adult lung, indicated by an increased mean linear intercept. Crtap expressions in the lung parenchyma in all pneumocytes.	Intrapulmonary: emphysematous changes
Dimori et al. (2020)	Crtap^−/−^ mice (number 18) and wild-type littermates (number 18). Lack of a functional prolyl 3-hydroxylation complex in the endoplasmic reticulum, which is essential for type I collagen post-translational modification and folding.	Model of recessive inherited OI forms.	Lung tissue histology.	Clear and dramatic enlargement of the acinar airspace and frequent loss of alveolar septa between all Crtap^−/−^ mice and wild-type littermates (at three months of age). Statistically significant difference of the linear intercept measurement.	Intrapulmonary: emphysematous changes, abnormal collagen synthesis
Thiele et al. (2012)	Aga2 mice severe (number = 6). Aga2 mice mild (number = 6), wild type (number = 6). Aga2: dominant frameshift mutation in the Col1a1 C-propeptide domain.	Model of OI II and III.	Lung tissue histology.	Aga2 severely affected lungs were haemorrhagic with alveolar bleeding, infiltrated with polymorphonuclear neutrophils and alveolar macrophages. Blood gas analysis revealed a reduction of 44% in arterial pO_2_ accompanied by a 61% decrease in oxygen saturation.	Intrapulmonary: abnormal collagen synthesis

pO_2_: partial pressure of oxygen.

Multiple lung abnormalities of the lung connective tissue in OI mice have been reported due to different causes (Table 1). Baglole et al. suggested a complex and multifaceted cause for respiratory complications, concluding that the distal airspace enlargement associated with emphysema is potentially a direct consequence of the dysfunctional type I collagen, which results in a loss of pulmonary elastic recoil [30]. Baldridge et al. concluded that lung defects in individuals with OI may be the primary result of abnormal collagen synthesis and not secondary to skeletal abnormalities [31]. Dimori et al. suggested that the collagen over modifications observed in CrtapKO mice and the potential resulting alterations in cross-linking of the collagen fibrils in OI may significantly weaken the strength of the alveolar wall and its ability to withstand the mechanical stress and strain that are typical of each breathing cycle.

This early rupture of the alveolar wall or developmental dysplasia of the lung parenchyma with defective alveolarization accounted for the alterations in the breathing pattern of the mice and altered respiratory mechanics [11,32].

### Extrapulmonary causes of abnormal lung function in OI patients

Extrapulmonary causes of abnormal lung function have been reported in eight studies of OI patients in whom the restrictive lung function pattern is related to the sternal, rib cage, or vertebral deformities (Table 2). Lo Mauro et al. studied the structural modifications of the rib cage and the breathing pattern of OI patients. A close relationship has been suggested between the restrictive respiratory pattern of OI and the severity of the disease, especially in OI type III in which sternal deformities appear to severely affect respiratory function. This is caused by the combination of pectus carinatum, brittle ribs, and spinal deformity that mechanically affect muscle function of the rib cage [8,33].

**Table 2.** Patients characteristics of studies supporting extrapulmonary causes of abnormal lung function in OI patients.

Authors	Patient phenotype	Age median (range) year	Type of assessment	Lung and chest abnormalities	Authors supported pathophysiology
LoMauro et al. (2012)	7 patients with OI III	26.1 (9.8–42.4)	Spirometry Opto-electronic plethysmography (rib cage geometry, breathing pattern, regional chest wall volume changes) Radiographic measurements	Altered breathing pattern in severe OI was present since childhood and it worsened with age. OI type III with by pectus carinatum and inspiratory paradoxical inward motion of the pulmonary rib cage, were associated with a high level of asynchrony between the three chest wall compartments.	Extra-pulmonary: pectus carinatum, brittle ribs, spinal deformity
	15 patients with OI IV	15.9 (4.5–27.3)			
	26 healthy controls	22.4 (4.1–40.7)			
LoMauro et al. (2018)	8 patients with OI III	5 (4.0–8.2)	Opto-electronic plethysmography Radiographic measurements	Patients with OI type III and IV showed decreased FVC and FEV1 compared to the predicted values. In the supine position, OI type III patients exhibited greater decrease in FVC due to pectus carinatum, paradoxical inspiratory inward motion of the pulmonary rib cage, significant thoraco-abdominal asynchrony and rib cage deformities.	Extra-pulmonary: pectus carinatum, brittle ribs, spinal deformity
	7 patients with OI I and IV	7 (6.5–8.0)			
	9 healthy controls	6.5 (5.0–8.0)			
Gimeno et al. (2019)	9 patients with OI III	41.0 (30.5–50.75)	Spirometry CT scans Geometric morphometrics in 3 D	Lower FEV1 and FVC values were observed in OI patients (males and females) with more horizontally aligned ribs, greater antero-posterior depth due to extreme transverse curve at rib angles and a strong spine invagination, greater asymmetry, and a shorter vertically, thoracic lumbar spine, which is relatively straight at levels 1–8 and shows marked kyphosis in the thoraco-lumbar junction. Regression analyses on the full sample showed a significant relation between rib shape and FEV1, FVC and FVC % predicted whereas thoracic spine shape was not related to any parameter.	Extra-pulmonary: horizontally aligned ribs
	3 patients with OI IV	41.0 (30.5–50.75)			
	12 healthy controls	64.5 (61–68.5)			
Wekre et al. (2013)	74 patients with OI I	45 (32–58)	Spirometry Radiographic measurements	Pulmonary compromise, as reflected in spirometry indices corrected with arm span height, revealed significant correlations to spinal deformities. Vertebral deformities were found in 67% of patients, most deformities were found in the mid-thoracic region. Scoliosis was found in 46%, nine patients exhibited torsion scoliosis.	Extra-pulmonary: spinal deformities
	9 patients with OI III	35 (28–42)			
	11 patients with OI IV	47 (40–54)			
Widmann et al. (1998)	15 OI patients (OI type undefined)	33.3 (24.8–41.8)	Spirometry Radiographic measurements Validated health self-assessment questionnaire	Thoracic scoliosis was strongly correlated with decreased predicted vital capacity. Significant diminution in the vital capacity below 50% occurred at an angle of 60°. Kyphosis and chest wall deformity were not predictive of decreased pulmonary function.	Extra-pulmonary: spinal deformities
Pan et al. (2006)	1 patient with OI IV	14	Spirometry Radiographic measurements Pedicle screw fixation technique (intervention)	Intervention (Table 3)	Extra-pulmonary: kyphoscoliosis
Kaplan et al. (2013)	4 patients with OI III with Thoracic Insufficiency Syndrome (TIS)	6, 7, 9, 11	Spirometry Radiographic measurements Expandable spinothoracic fixation device (intervention)	Intervention (Table 3)	Extra-pulmonary: pectus carinatum, brittle ribs, spinal deformity
Falvo et al. (1973)	11 patients (4 severe OI, 7 mild OI)	13.5 (4–34)	Standard Spirometry Radiographic measurements Blood gas measurements?	Reduction of VC and increase in RV and RV/TLC ratio were found only in patients with kyphoscoliosis. Other parameters of pulmonary function were within normal limits. No patient had severe hypoxaemia or hypercapnea.	Extra-pulmonary: pectus carinatum, brittle ribs, spinal deformity

FEV1: volume in 1 s; FVC: forced vital capacity; VC: vital capacity; RV: residual volume; TLC: total lung capacity.

Wekre et al. and Widmann et al. both support that compromised OI pulmonary function shows a significant correlation with spinal deformities, especially with thoracic scoliosis [12,13]. Instead, Gimeno et al. found that decreased respiratory function in OI patients is related to an anatomic configuration characterized by more horizontally aligned ribs, which indicates that the alteration of the ventilation largely depends on the position of the ribs rather than the shape of the spine [34]. One study also showed the absence of abnormalities in ventilation-perfusion relationships based on arterial blood gas measurements [7]. Table 3 shows two reports of thoracic elongation and surgery which improved pulmonary function in OI type IV patients, implicitly supporting the extrinsic role of thoracic malformations in lung function disorders [35,36].

**Table 3.** Lung and chest abnormalities after surgery.

Authors	Patient phenotype	Intervention	Lung and chest abnormalities after intervention
Pan et al. (2006)	1 patient with OI IV	Correction of severe kyphoscoliosis using the 3-rod all pedicle screw fixation technique.	The predicted forced vital capacity predicted forced expiratory volume in 1 s and vital capacity of the lung of the patient had improved 2-fold. The Cobb angles of scoliosis and thoracic kyphosis were corrected from 110 to 68° and from 107 to 39°.
Kaplan et al. (2013)	4 patients with OI III with Thoracic Insufficiency Syndrome (TIS)	Patients were treated with a novel expandable spinothoracic fixation device.	The mean Cobb angle improved to 32% in the coronal plane. Pulmonary function improved in all patients, with mean increases of 45% in forced vital capacity, 93% in forced expiratory flow, and 43% in pO_2_, PaCO_2_ decreased an average of 30% and returned to normal values.

pO_2_: partial pressure of oxygen; PaCO_2_: partial pressure of carbon dioxide.

### Observational studies and intrinsic lung disease

Table 4 offers an overview of the lung function in relation to chest abnormalities in OI patients, described in seven studies that support the hypothesis that impaired lung function is intrinsic to OI. This corroborates the findings in the OI mouse models, demonstrating post-natal primary defects in the lung parenchyma. Abnormal or insufficient collagen type I was proposed as the underlying cause of the pulmonary disease. Bronheim et al. found no correlation between the largest spinal curve and the FEV_1_/FVC ratio in 30 patients, supporting that decreased respiratory function may be an intrapulmonary consequence of altered collagen type I [37]. Khan et al. also failed to detect an association between decreased pulmonary function and the severity of scoliosis, suggesting that restrictive lung function is caused by factors inherent in OI and not exclusively by thoracic cage deformities [38]. Thiele et al. suggested that type I collagen alterations directly cause pathological changes in heart and lung tissue and that the physiopathology of lung disease is independent of skeletal dysplasia [11].

**Table 4.** Patients characteristics of the studies supporting intrapulmonary causes of abnormal lung function in OI patients.

Authors	Patient phenotype	Age	Type material	Lung and chest abnormalities	Authors supported pathophysiology
Bronheim et al. (2019)	3 patients with OI I	27.6 (12.9–42.3)	Spirometry Radiographic measurements	Restrictive lung function in 83% of the patients, pulmonary comorbidity like asthma or chronic obstructive pulmonary disease was in 40% of individuals. Scoliosis was present in 25 individuals (83.3%), 4 of whom (13.3%) had undergone corrective surgery. No correlation was detected between the largest curve and FEV1/FVC1 ratio. No significant correlation between scoliosis and pulmonary function parameters.	Intra-pulmonary: intrinsic consequence of altered collagen type I
	10 patients with OI III				
	14 patients with OI IV				
	1 patient with OI VIII				
	2 patients with OI IX				
Khan et al. (2020)	9 patients with OI I	39 (19–67)	Spirometry Radiographic measurements ECG, echocardiogram Quality-of-life assessments	Seventy percent (21 of 30) of the population had scoliosis. Seventy-seven percent of participants (23 of 30) had restrictive lung function. Forty-three percent (13 of 30) of the study participants had lower total lung capacity than the reported reference value FEV1/FVC did not correlate with scoliosis curve magnitude. Twenty-five of 29 individuals had bronchial wall thickening, 16 had ground-glass opacities, 12 had atelectasis, 11 had scarring, and five had mosaic air trapping.	Intra-pulmonary: intrinsic consequence of altered collagen type I
	8 patients with OI III				
	12 patients with OI IV				
Morikawa et al. (2016)	1 patient with OI (unspecified)	19	Radiographic measurements CT scan Lung histology	Diffuse reticular shadows on chest X-ray images Diffuse reticulonodular shadows in both lungs Old and recent haemorrhagic areas and emphysematous change around bone formation were observed by stereomicroscopy	Intra-pulmonary: pulmonary ossifications with fibrosis
SHAPIRO et al. (1989)	1 patient with OI II	1 day	Lung histology Fibroblast culture	Histopathologic examination of the lungs showed a marked decrease in the quantity of parenchyma in relation to hilar structures. Alveolar number per acinus was reduced. No bronchopneumonia or hyaline membrane formation was observed.	Intra-pulmonary: defective pro-alpha 1(I) synthesis
Thibeault et al. (1995)	1 patient with OI II	1 day	Lung histology	The volume density of the combined alveolar and alveolar duct spaces and parenchymal septal tissue in the OI II lung was increased compared with the control lungs. The absolute number of conducting airway generations and the proportion and number of terminal bronchioles were similar in both infants. The proportion of respiratory bronchioles and the number per unit area were lower in the OI II, suggesting that each acinus had fewer saccular clusters.	Intra-pulmonary: intrinsic consequence of altered collagen type I
	1 patient healthy control				
Himakhun et al. (2012)	1 patient with OI II	6 day	Lung histology	Decrease in size of both lungs. Histology showing immature lungs with congestion and hyaline membrane, compatible with clinically diffuse alveolar damage and respiratory failure.	Intra-pulmonary: intrinsic consequence of altered collagen type I
Thiele et al. (2012)	23 patients with OI III	9.6 (3–18)	Spirometry Radiographic measurements ECG, echocardiograms	Thirty six of 46 children and young adults in the study group (78.3%) developed scoliosis >10*, with mean curvature ≈25*. OI patients with scoliosis have progressive decline of forced vital capacity (FVC), tidal lung capacity (TLC) and vital capacity (VC) with worsening scoliosis. Pulmonary function parameters decline significantly with age for all OI patients, including lung volumes and flow rates. The decline in FVC, TLC and VC with age was significantly greater for type III than the milder type IV OI patients. Although scoliosis contributes to pulmonary function test decline in OI, significant decline occurs in the absence of scoliosis. Lung function declined significantly in 20 participants who had minimal scoliosis (≤10* curvature).	Intra-pulmonary: intrinsic consequence of altered collagen type I
	23 patients with OI IV				

FEV1: volume in 1 s; FVC: forced vital capacity; VC: vital capacity; RV: residual volume; TLC: total lung capacity.*Degree.

Three case reports described histological analysis of lung autopsy findings in OI type II patients, suggesting a possible role for defective procollagen type I synthesis in pulmonary airway development [19,20,22]. Morikawa et al. reported an interesting case of a 19-year-old OI patient with diffuse pulmonary ossification which probably occurred during repair of alveolar damage due to decreased collagen type I production [39].

## Discussion

Respiratory failure is a major cause of death in patients with Osteogenesis Imperfecta, one of the most common inherited connective tissue disorders. Acute or chronic respiratory failure has been correlated to the severity of the OI disease [14,15,17]. However, the underlying mechanism and pathophysiology have received relatively little attention. Little is known about whether and how OI can affect intrapulmonary tissues beyond its known effect on the thoracic skeleton. Research and treatment for OI have almost been exclusively focussed on fracture prevention, correction of bone deformities while other complications, such as hearing loss, dental problems, and pain relief are rarely addressed. Respiratory problems seem to receive particular attention only at a late stage when they have become a clinical problem. As a result, to date, there are no established international protocols for follow-up pulmonary surveillance or treatment of pulmonary manifestations in patients with OI.

To our knowledge, this is the first systematic review to examine the hypothesis that the cause of lung disease in OI may extend beyond thoracic skeleton deformities. This review identifies different potential causes of respiratory failure in OI and presents a new concept and perspective for care and research on pulmonary problems in OI. This review shows that in OI mice, changes in the lung parenchyma are specifically indicative of emphysematous changes [11,30–32]. These mice studies described enlarged alveolar airspaces, that may be due to either destruction of lung tissue (emphysema in the stricter sense) or abnormal growth of lung tissue (a kind of dysplasia). These defects have been attributed to the compromised type I collagen which consists of a major tissue component of mammalian lungs, found in bronchi, blood vessels, and within the alveolar interstitium [28,40]. Collagen type I is also known to be fundamental to the maintenance of lung structure and function due to its ability to effectively respond to mechanical stress during ventilation [26,28,40,41]. However, these preclinical studies are not exempt from limitations. These studies represent a limited number of OI types and genetic causes, thus findings from these models cannot be extrapolated to every type of OI. In particular, the Col1a1Jrt/+ model simulates severe dominant OI, Crtap knockout mice are a model of recessive OI and Aga2 mice recapitulate OI type II and III. Nevertheless, these four animal studies reveal valuable insight in lung histology indicating that the affected primary lung parenchyma defect in OI may play a greater role than previously suspected.

The presented studies reveal a dichotomy in the perspective and findings of the reported studies in patients. Eight studies reported that altered respiratory function in OI is related to the anatomic configuration and the severity of the disease [7,8,12,13,33–36]. These studies show a clear correlation between thoracic malformations and pulmonary function parameters but did not rule out a concomitant intrapulmonary cause of the lung disease. On the contrary, there are also seven clinical studies supporting the hypothesis that impaired lung function is intrinsic to OI, meaning that altered type I collagen can play a crucial role in the alteration of the interstitium of the lung parenchyma, leading to restrictive or obstructive disease. These clinical studies are restricted by the small number of participants and the limited number of histological examinations that support the hypothesis of altered lung parenchyma. However, the post-mortem biopsy described in various case reports of severe human OI type II support that patient death was caused by reduced lung growth, diffuse alveolar damage, and immature acinar development.

We support that the underlying collagen defect plays a crucial role in the development and organization of the lung parenchyma in patients with OI, especially in patients with a structural alteration of type I collagen (OI type III and IV). However molecular diagnosis of patients was not performed in these studies which may help to ascertain the type of collagen defect. Interestingly, all examined OI mouse models with primary defects in the lung parenchyma shared structural alterations in collagen type I. We await future studies to show if that is also the case in cases of only reduced collagen production (less severe OI type I).

Many clinical studies used lung function examination as the main outcome. Patients with OI type I can have a normal lung function when the arm span is used to calculate the predicted values [9,12]. In type III and IV, the restriction is often found in both children and adults. The restriction is either proven by a low TLC or suggested by a reduced (F)VC or high FEV_1_/FVC (the latter denoted as ‘restrictive physiology’) (Tables 2 and 4). Limited data on the RV show a normal or a slightly decreased RV in most subjects [7,8,13]. Only in some patients, with kyphoscoliosis, the RV was increased. Data on the FRC (functional residual capacity) are also limited. In one of the larger studies, the FRC was reduced in type III and IV (mean values 61 and 75% of predicted, respectively) [8]. The transfer factor of the lung for CO (TLCO) was found to be either normal or increased [38]. In addition to chest wall restriction, lung function may be affected by intrapulmonary factors, such as ground-glass opacities, atelectasis, or scarring that have been observed by CT-scan [38]. In the latter study, emphysema was only found in one out of 29 patients (in the form of paraseptal emphysema). On the other hand, studies in mice indicate that emphysema can occur in OI (Table 1). Emphysema was also found in the lung tissue of deceased neonates with type II disease (Table 4). The histology of the 19-year-old OI patient who underwent VATS also showed emphysematous changes, along with haemorrhagic areas and dendritic-shaped ossifications (Table 4). We, therefore, suggest that the occurrence of emphysema is possible in patients with OI which necessitates the use of more test and histological examinations (Supplementary Figure 2).

The question remains whether emphysematous changes are compatible with the restrictive lung function that is mostly found in patients with advanced disease. Emphysema tends to enlarge the lung volume because of high lung compliance [42]. We suppose that this effect can be masked by a low chest wall compliance, especially in the case of severe scoliosis, although kyphosis or other chest wall deformities may also contribute [43].

Our expert recommendation, based on our review assessment, is the monitoring of respiratory functions by measuring both spirometric parameters (including ERV) and the single-breath CO-transfer with Helium dilution; this is suggested at the initial assessment and the yearly follow-ups of patients with qualitative collagen alterations, and every 3 years in patients with quantitative collagen alterations. Spirometry in patients with thoracic deformities or in patients who are unable to stand should be corrected for body height and arm span height. In patients with type III and IV also the spine curvature and the chest wall deformities should be carefully monitored. To prevent serious respiratory complications in OI patients, difficulties in breathing and respiratory infections should be treated immediately, and patients should be regularly vaccinated against influenza. Our recommendation also includes monitoring of the patients by a multidisciplinary team, including a pulmonologist and cardiologist [44].

In conclusion, pulmonary function in OI patients should be carefully monitored even in the absence of sternal, rib cage, or vertebral deformities. Reduced lung function may deteriorate in relation to the rib cage or vertebral deformities but this is likely not the only cause of pulmonary disease in OI patients. Type I collagen defects may have a crucial role in the development of lung parenchyma in OI patients, leading to lung disease. Spirometric parameters should always be corrected based on height and complemented with the examination of lung physiology. Further research on this topic is needed to improve knowledge of the pathogenesis of OI pulmonary alterations to identify patient risk groups and suitable therapeutic targets to optimize clinical management for the prevention of morbidity and mortality in OI.

## Supplementary Material

Supplemental Material

## Data Availability

There is no raw data associated with this systematic review. The authors confirm that the data supporting the findings of this study are available within the article and its supplementary materials.

## References

[1] Sillence DO, Senn A, Danks DM. Genetic heterogeneity in osteogenesis imperfecta. J Med Genet. 1979;16(2):101–116. 458828 10.1136/jmg.16.2.101 PMC1012733

[2] van Dijk FS, Cobben JM, Kariminejad A, et al. Osteogenesis imperfecta: a review with clinical examples. Mol Syndromol. 2011;2(1):1–20. 22570641 10.1159/000332228 PMC3343766

[3] Van Dijk FS, Sillence DO. Osteogenesis imperfecta: clinical diagnosis, nomenclature and severity assessment. Am J Med Genet A. 2014;164(6):1470–1481. 10.1002/ajmg.a.36545 PMC4314691 24715559

[4] Monti E, Mottes M, Fraschini P, et al. Current and emerging treatments for the management of osteogenesis imperfecta. Ther Clin Risk Manag. 2010;6:367–381. 20856683 10.2147/tcrm.s5932 PMC2940745

[5] Marini JC, Forlino A, Bächinger HP, et al. Osteogenesis imperfecta. Nat Rev Dis Primers. 2017;3:17052. 28820180 10.1038/nrdp.2017.52

[6] Forlino A, Marini JC. Osteogenesis imperfecta. Lancet. 2016;387(10028):1657–1671. 26542481 10.1016/S0140-6736(15)00728-X PMC7384887

[7] Falvo KA, Klain DB, Krauss AN, et al. Pulmonary function studies in osteogenesis imperfecta. Am Rev Respir Dis. 1973;108(5):1258–1260. 4746589 10.1164/arrd.1973.108.5.1258

[8] LoMauro A, Pochintesta S, Romei M, et al. Rib cage deformities alter respiratory muscle action and chest wall function in patients with severe osteogenesis imperfecta. PLOS One. 2012;7(4):e35965. 22558284 10.1371/journal.pone.0035965 PMC3338769

[9] Takken T, Terlingen HC, Helders PJ, et al. Cardiopulmonary fitness and muscle strength in patients with osteogenesis imperfecta type I. J Pediatrics. 2004;145(6):813–818. 10.1016/j.jpeds.2004.08.003 15580207

[10] Tam A, Chen S, Schauer E, et al. A multicenter study to evaluate pulmonary function in osteogenesis imperfecta. Clin Genet. 2018;94(6):502–511. 30152014 10.1111/cge.13440 PMC6235719

[11] Thiele F, Cohrs CM, Flor A, et al. Cardiopulmonary dysfunction in the osteogenesis imperfecta mouse model Aga2 and human patients are caused by bone-independent mechanisms. Hum Mol Genet. 2012;21(16):3535–3545. 22589248 10.1093/hmg/dds183 PMC3406754

[12] Wekre LL, Kjensli A, Aasand K, et al. Spinal deformities and lung function in adults with osteogenesis imperfecta. Clin Respir J. 2014;8(4):437–443. 24308436 10.1111/crj.12092

[13] Widmann RF, Bitan FD, Laplaza FJ, et al. Spinal deformity, pulmonary compromise, and quality of life in osteogenesis imperfecta. Spine. 1999;24(16):1673–1678. 10472101 10.1097/00007632-199908150-00008

[14] McAllion SJ, Paterson CR. Causes of death in osteogenesis imperfecta. J Clin Pathol. 1996;49(8):627–630. 8881910 10.1136/jcp.49.8.627 PMC500603

[15] Folkestad L, Hald JD, Canudas-Romo V, et al. Mortality and causes of death in patients with osteogenesis imperfecta: a register-based nationwide cohort study. J Bone Miner Res. 2016;31(12):2159–2166. 27345018 10.1002/jbmr.2895

[16] Yonko EA, Emanuel JS, Carter EM, et al. Respiratory impairment impacts QOL in osteogenesis imperfecta independent of skeletal abnormalities. Arch Osteoporos. 2020;15(1):153. 33009598 10.1007/s11657-020-00818-0

[17] Folkestad L. Mortality and morbidity in patients with osteogenesis imperfecta in Denmark. Danish Med J. 2018;65(4):1–51. 29619932

[18] Ayadi ID, Hamida EB, Rebeh RB, et al. Perinatal lethal type II osteogenesis imperfecta: a case report. Pan Afr Med J. 2015;21:11. 26401205 10.11604/pamj.2015.21.11.6834 PMC4561136

[19] Shapiro JR, Burn VE, Chipman SD, et al. Pulmonary hypoplasia and osteogenesis imperfecta type II with defective synthesis of alpha I(1) procollagen. Bone. 1989;10(3):165–171. 2803853 10.1016/8756-3282(89)90049-5

[20] Thibeault DW, Pettett G, Mabry SM, et al. Osteogenesis imperfecta type IIA and pulmonary hypoplasia with normal alveolar development. Pediatr Pulmonol. 1995;20(5):301–306. 8903902 10.1002/ppul.1950200508

[21] Barros CA, Rezende Gde C, Araujo Júnior E, et al. Prediction of lethal pulmonary hypoplasia by means fetal lung volume in skeletal dysplasias: a three-dimensional ultrasound assessment. J Matern Fetal Neonat Med. 2016;29(11):1725–1730. 10.3109/14767058.2015.1064887 26135769

[22] Himakhun W, Rojnueangnit K, Prachukthum S. Perinatal lethal osteogenesis imperfecta in a Thai newborn: the autopsy and histopathogical findings. J Med Assoc Thai. 2012;95:S190–S194. 23964465

[23] Pierce JA, Hocott JB, Ebert RV. Studies of lung collagen and elastin. Am Rev Respir Dis. 1959;80(1 Part 2):45–49. 13670403 10.1164/arrd.1959.80.1P2.45

[24] Pierce JA, Hocott JB. Studies on the collagen and elastin content of the human lung. J Clin Invest. 1960;39(1):8–14. 14432825 10.1172/JCI104030 PMC290656

[25] Bradley KH, McConnell SD, Crystal RG. Lung collagen composition and synthesis. Characterization and changes with age. J Biol Chem. 1974;249(9):2674–2683. 4364025

[26] Laurent GJ. Lung collagen: more than scaffolding. Thorax. 1986;41(6):418–428. 3024347 10.1136/thx.41.6.418 PMC460358

[27] Burgeson RE, Nimni ME. Collagen types. Molecular structure and tissue distribution. Clin Orthop Relat Res. 1992;282:250–272. 1516320

[28] Bienkowski RS, Gotkin MG. Control of collagen deposition in mammalian lung. Proc Soc Exp Biol Med. 1995;209(2):118–140. 7770462 10.3181/00379727-209-43886a

[29] Murad MH, Sultan S, Haffar S, et al. Methodological quality and synthesis of case series and case reports. BMJ Evid Based Med. 2018;23(2):60–63. doi:10.1136/bmjebm-2017-110853. 29420178 PMC6234235 29420178

[30] Baglole CJ, Liang F, Traboulsi H, et al. Pulmonary and diaphragmatic pathology in collagen type I α1 mutant mice with osteogenesis imperfecta. Pediatr Res. 2018;83(6):1165–1171. 29538357 10.1038/pr.2018.36

[31] Baldridge D, Lennington J, Weis M, et al. Generalized connective tissue disease in Crtap^−/−^ mouse. PLOS One. 2010;5(5):e10560. 20485499 10.1371/journal.pone.0010560 PMC2868021

[32] Dimori M, Heard-Lipsmeyer ME, Byrum SD, et al. Respiratory defects in the CrtapKO mouse model of osteogenesis imperfecta. Am J Physiol Lung Cell Mol Physiol. 2020;318(4):L592–L605. 32022592 10.1152/ajplung.00313.2019 PMC7191481

[33] LoMauro A, Fraschini P, Pochintesta S, et al. Ribcage deformity and the altered breathing pattern in children with osteogenesis imperfecta. Pediatr Pulmonol. 2018;53(7):964–972. 29766672 10.1002/ppul.24039

[34] Sanchis-Gimeno JA, Lois-Zlolniski S, María González-Ruiz J, et al. Association between ribs shape and pulmonary function in patients with Osteogenesis Imperfecta. J Adv Res. 2020;21:177–185. 32071786 10.1016/j.jare.2019.10.007 PMC7015465

[35] Pan CH, Ma SC, Wu CT, et al. All pedicle screw fixation technique in correcting severe kyphoscoliosis in an osteogenesis imperfecta patient: a case report. J Spinal Disord Tech. 2006;19(5):368–372. 16826011 10.1097/01.bsd.0000208253.06706.42

[36] Kaplan L, Barzilay Y, Hashroni A, et al. Thoracic elongation in type III osteogenesis imperfecta patients with thoracic insufficiency syndrome. Spine. 2013;38(2):E94–E100. 23138403 10.1097/BRS.0b013e31827a7566

[37] Bronheim R, Khan S, Carter E, et al. Scoliosis and cardiopulmonary outcomes in osteogenesis imperfecta patients. Spine. 2019;44(15):1057–1063. 31335789 10.1097/BRS.0000000000003012

[38] Khan SI, Yonko EA, Carter EM, et al. Cardiopulmonary status in adults with osteogenesis imperfecta: intrinsic lung disease may contribute more than scoliosis. Clin Orthopaed Relat Res. 2020;478:2833–2843. 10.1097/CORR.0000000000001400 PMC7899416 32649370

[39] Morikawa M, Fukuda Y, Terasaki Y, et al. Osteogenesis imperfecta associated with dendriform pulmonary ossification. Am J Respir Crit Care Med. 2016;193(4):460–461. 26431159 10.1164/rccm.201505-0942IM

[40] Madri JA, Furthmayr H. Collagen polymorphism in the lung. An immunochemical study of pulmonary fibrosis. Hum Pathol. 1980;11(4):353–366. 6997183 10.1016/s0046-8177(80)80031-1

[41] Patino MG, Neiders ME, Andreana S, et al. Collagen: an overview. Implant Dent. 2002;11(3):280–285. 12271567 10.1097/00008505-200207000-00014

[42] Gibson GJ, Pride NB, Davis J, et al. Exponential description of the static pressure-volume curve of normal and diseased lungs. Am Rev Respir Dis. 1979;120(4):799–811. 315738 10.1164/arrd.1979.120.4.799

[43] Koumbourlis AC. Scoliosis and the respiratory system. Paediatr Respir Rev. 2006;7(2):152–160. 16765303 10.1016/j.prrv.2006.04.009

[44] Eekhoff EMW, Micha D, Forouzanfar T, et al. Collaboration around rare bone diseases leads to the unique organizational incentive of the Amsterdam Bone Center. Front Endocrinol. 2020;11:481. 10.3389/fendo.2020.00481 PMC7431598 32849274

