# Supplementary material for: Pathophysiology of respiratory failure in patients with osteogenesis imperfecta: a systematic review
Source: Ann Med. 2021 Sep 27;53(1):1676–87. doi: 10.1080/07853890.2021.1980819 (PMC8477932; doi:10.1080/07853890.2021.1980819)

SUPPLEMENTAL MATERIAL

**Interpretation of lung function in OI**

Emphysema tends to enlarge the lung volume because of a high lung compliance. (1)

We suppose that this effect can be masked by a low chest wall compliance, especially in the case of severe scoliosis, although kyphosis or other chest wall deformities may also contribute. (2)

See Figure 2, which shows a hypothetical Campbell diagram for a healthy subject and a patient with OI (in the left and right panel, respectively) (3). In this diagram, the external volume of the lung (*L*) and the internal volume of the chest wall (*W*) are plotted against the mean pleural pressure. These are static pressure-volume relations which are mainly determined by elastic forces (and to a lesser extent by the weight of the tissues). At a given volume, the compliance is the slope of the tangent to the curve (to be precise, the absolute value thereof). The lower the compliance, the flatter the curve. At the intersection point of the two curves, the inward elastic forces of the lung are in equilibrium with the outward elastic forces of the chest wall. In a healthy subject (Figure 2A), this occurs at a pressure of −3.5 cmH_2_O. At this underpressure in the pleural space, the lung is stretched to 1.5 L, while chest wall is contracted to the same volume. This volume is the FRC. The hatched area depicts the elastic work of breathing at inhalation of 1 liter of air from FRC. In the patient with OI (Figure 2B), the compliance of the chest wall is reduced, i.e., the pressure-volume curve *W* is flatter. The intersection point now occurs at a lower lung volume, meaning that the resting volume (the FRC) is smaller. The elastic work needed to inhale 1 liter of air is now considerably greater. The elastic component of the work of breathing, given by the triangular area between the two curves, progressively increases at deeper inhalations. This makes deep inhalations almost impossible. The total work of breathing, also including resistive work, is even higher when the airways are narrowed.

Figure 2B shows the consequences of reduced lung compliance (curve *L*_1_). In OI, the compliance of the lung can be reduced by atelactasis, scarring, or ossification of lung tissue. Part of these alterations can be directly attributed to the loss of collagen fibers in the lung. Recurrent inflammations, with or without infection, may also be involved. In addition, scoliosis itself can reduce the compliance of the lung (4, 5). The same holds for other causes of chest wall restriction, as appears from studies on the effects of chest strapping in healthy subjects (6, 7). As seen in Figure 2B, a reduced lung compliance gives a further reduction in the FRC and a further augmentation in the elastic work of breathing that is needed to inhale 1 liter of air (also including the horizontally hatched area). However, an increase in lung compliance, as can be expected in advanced emphysema (curve *L*_2_), still results in a low FRC due to the low compliance of the chest wall. The elastic work of breathing is less than in the case of reduced lung compliance, but inhalation of large volumes is still impossible because of the elastic forces of the chest wall. Thus, a flaccid lung placed in a rigid chest cage still results in a restrictive lung function. It is also possible that the lung compliance in OI is only reduced at high lung volumes, as suggested by the mouse model of Dimori et al. (2019). See curve *L*_3_ in Figure 2B. A possible explanation is that collagen is mainly stretched at high volumes, whereas elastin can already induce elastic forces at low lung volumes (8, 9). In this respect, enlargement of alveolar spaces in OI differs from the common type of emphysema, which is mainly due to loss of elastin instead of collagen (8).

When the RV is normal or only slightly reduced in patients with OI, the lower FRC leads to a lower ERV (expiratory reserve volume, the difference between FRC and RV). As illustrated in Figure 2B, a low ERV can be a direct consequence of low chest wall compliance and indicates high elastic work of breathing. The ERV is further reduced when the lung compliance is reduced as well, or when the RV is increased (as occurs in some patients with kyphoscoliosis). From the data of LoMauro et al. (2012), it can be derived that the mean ERV was 0.13 l in type III and 0.45 l in type IV. This amounts to respectively 11% and 19% of the mean FVC, which is indeed relatively low. In OI, the ERV may be interpreted as a parameter of low chest wall compliance due to skeletal malformation, especially when obesity is not involved (10). The practical significance of the ERV is that it can be measured by spirometry, without the need for body plethysmography or other techniques to measure the lung volume.

**References**

1. Gibson GJ, Pride NB, Davis J, Schroter RC. Exponential description of the static pressure-volume curve of normal and diseased lungs. Am Rev Respir Dis. 1979;120(4):799-811. Epub 1979/10/01. doi: 10.1164/arrd.1979.120.4.799. PubMed PMID: 315738.

2. Koumbourlis AC. Scoliosis and the respiratory system. Paediatr Respir Rev. 2006;7(2):152-60. Epub 2006/06/13. doi: 10.1016/j.prrv.2006.04.009. PubMed PMID: 16765303.

3. Roussos C CE, , Fishman AP, Macklem PT, Mead J (editors). Respiratory muscle energetics. In: Handbook of Physiology part III: Mechanics of breathing Handbook of Physiology. 1986.

4. Cooper DM, Rojas JV, Mellins RB, Keim HA, Mansell AL. Respiratory mechanics in adolescents with idiopathic scoliosis. Am Rev Respir Dis. 1984;130(1):16-22. Epub 1984/07/01. doi: 10.1164/arrd.1984.130.1.16. PubMed PMID: 6742606.

5. Katsaris G, Loukos A, Valavanis J, Vassiliou M, Behrakis PK. The immediate effect of a Boston brace on lung volumes and pulmonary compliance in mild adolescent idiopathic scoliosis. European spine journal : official publication of the European Spine Society, the European Spinal Deformity Society, and the European Section of the Cervical Spine Research Society. 1999;8(1):2-7. Epub 1999/04/06. doi: 10.1007/s005860050119. PubMed PMID: 10190847; PubMed Central PMCID: PMCPMC3611133.

6. Klineberg PL, Rehder K, Hyatt RE. Pulmonary mechanics and gas exchange in seated normal men with chest restriction. Journal of applied physiology: respiratory, environmental and exercise physiology. 1981;51(1):26-32. Epub 1981/07/01. doi: 10.1152/jappl.1981.51.1.26. PubMed PMID: 7263421.

7. Scheidt M, Hyatt RE, Rehder K. Effects of rib cage or abdominal restriction on lung mechanics. Journal of applied physiology: respiratory, environmental and exercise physiology. 1981;51(5):1115-21. Epub 1981/11/01. doi: 10.1152/jappl.1981.51.5.1115. PubMed PMID: 7298451.

8. Gefen A, Elad D, Shiner RJ. Analysis of stress distribution in the alveolar septa of normal and simulated emphysematic lungs. Journal of biomechanics. 1999;32(9):891-7. Epub 1999/08/25. doi: 10.1016/s0021-9290(99)00092-5. PubMed PMID: 10460125.

9. Laurent GJ. Lung collagen: more than scaffolding. Thorax. 1986;41(6):418-28. Epub 1986/06/01. doi: 10.1136/thx.41.6.418. PubMed PMID: 3024347; PubMed Central PMCID: PMCPMC460358.

10. Jones RL, Nzekwu MM. The effects of body mass index on lung volumes. Chest. 2006;130(3):827-33. Epub 2006/09/12. doi: 10.1378/chest.130.3.827. PubMed PMID: 16963682.

## Figure Legends

Figure 2. Schematic pressure-volume relations for lung (*L*, blue) and chest wall (*W*, green) in a healthy subject (A) and a patient with Osteogenesis Imperfecta, with reduced chest wall compliance (B). TLC, total lung capacity; FRC, functional residual capacity; RV, residual volume; ERV, expiratory reserve volume. In both panels, the hatched area (oblique lines) represents the elastic work of breathing needed to inhale 1 liter of air from FRC, assuming that the lung compliance is normal. In Figure 2B, the horizontally hatched area represents the additional work of breathing if the lung compliance is reduced. *L*_1_, *L*_2_, and *L*_3_, pressure-volume curves for the lung, with respectively reduced compliance, increased compliance, and increased compliance at high lung volumes only.

**Figure 2.**


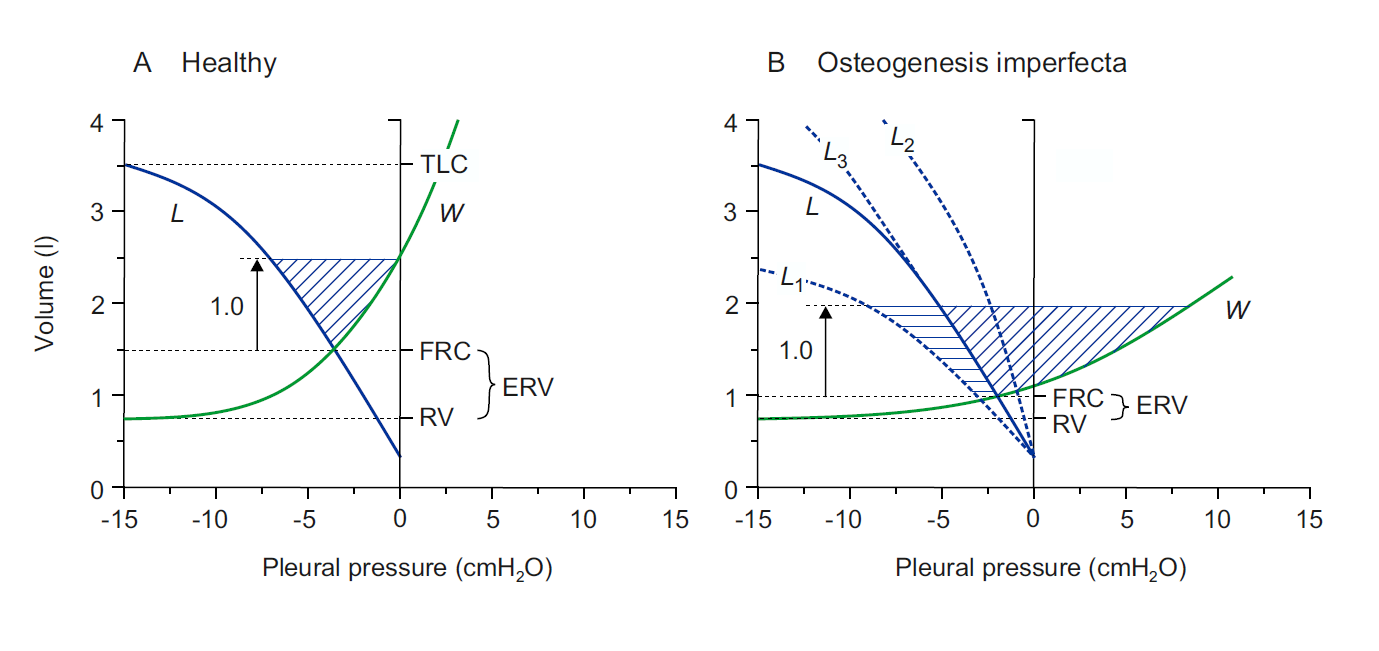

Supplement: Supplemental Material [file IANN_A_1980819_SM4126.docx]
